# Osteopontin regulates type I collagen fibril formation in bone tissue^☆^

**DOI:** 10.1016/j.actbio.2020.04.040

**Published:** 2021-01-15

**Authors:** Baptiste Depalle, Catriona M. McGilvery, Sabah Nobakhti, Nouf Aldegaither, Sandra J. Shefelbine, Alexandra E. Porter

**Affiliations:** a Department of Materials Science and Engineering, Imperial College London, London, United Kingdom; b The Forsyth Institute, Cambridge, MA United States; c Department of Mechanical and Industrial Engineering, Northeastern University, Boston, MA United States; d Department of Bioengineering, Northeastern University, Boston, MA United States; e College of Science and Health Professions, King Saud bin Abdulaziz University for Health Sciences, Riyadh, Saudi Arabia

**Keywords:** Osteopontin, Non-collagenous proteins, Bone, Mineralization, Collagen, Scanning electron nano beam diffraction

## Abstract

Osteopontin (OPN) is a non-collagenous protein involved in biomineralization of bone tissue. Beyond its role in biomineralization, we show that osteopontin is essential to the quality of collagen fibrils in bone. Transmission electron microscopy revealed that, in *Opn^−/−^* tissue, the organization of the collagen fibrils was highly heterogeneous, more disorganized than WT bone and comprised of regions of both organized and disorganized matrix with a reduced density. The *Opn^−/−^* bone tissue also exhibited regions in which the collagen had lost its characteristic fibrillar structure, and the crystals were disorganized. Using nanobeam electron diffraction, we show that damage to structural integrity of collagen fibrils in *Opn^−/-^* bone tissue and their organization causes mineral disorganization, which could ultimately affect its mechanical integrity.

**Statement of Significance:**

This study presents new evidence about the role of osteopontin (OPN) – a non-collagenous protein – on the structure and organization of the organic and mineral matrix in bone. In previous work, osteopontin has been suggested to regulate the nucleation and growth of bone mineral crystals and to form sacrificial bonds between mineralized collagen fibrils to enhance bone's toughness. Our findings show that OPN plays a crucial role before mineralization, during the formation of the collagen fibrils. OPN-deficient bones present a lower collagen content compared to wild type bone and, at the tissue level, collagen fibrils organization can be significantly altered in the absence of OPN. Our results suggest that OPN is critical for the formation and/or remodeling of bone collagen matrix. Our findings could lead to the development of new therapeutic strategies of bone diseases affecting collagen formation and remodeling.

## Graphical abstract

**Figure:**
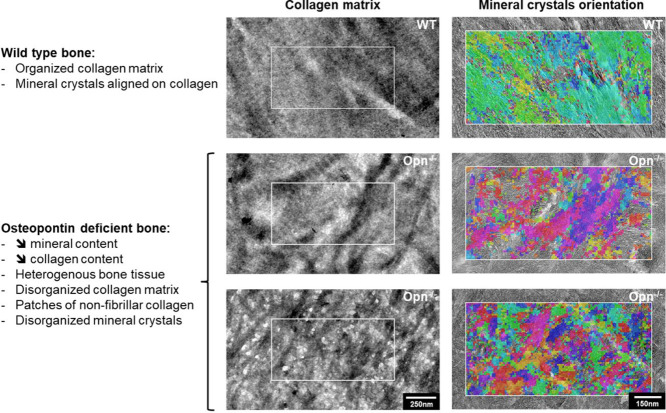

## 1 Introduction

Bone exhibits remarkable mechanical properties that can be attributed to its complex nanocomposite structure. At the nanoscale, bone is made of an organic matrix, filled by a mineral phase and water [1,2]. The organic matrix comprises around 25% of bone weight and consists of about 90% of type I collagen molecules assembled into fibrils. The remaining matrix is composed of non-collagenous proteins (NCPs). The mineral phase comprises, on average, 65% of hydrated bone weight and is made up of nanometer-sized apatite platelets.

Traditionally, the study of NCPs was limited to their role as signaling molecules in biological processes, including bone formation, resorption, and turnover. More recently, several studies revealed that NCPs are likely to be an integral component of the structural integrity of bones’ matrix and play a critical role in the mechanical behavior of tissues [3], [4], [5]. For example, the amount of NCPs is considerably reduced in osteoporotic bone matrix, which could be an origin of osteoporotic bone's fragility [6]. A more comprehensive understanding of the role of NCPs in influencing bone quality may improve both the prediction and prevention of fracture risk [7].

Among NCPs, osteopontin (OPN), which represents 1–2% of bone's NCPs [8], is hypothesized to play a major role in bone's tissue structure and mechanics. Since OPN is a highly charged and phosphorylated protein and has a high affinity to calcium, it has been attributed multiple functional roles in the bioregulation of bone mineral [9], [10], [11]. However, although OPN has also been shown to affect collagen mineralization *in vitro* [12], most of the studies on OPN-deficient mice have failed to indicate any major defect in mineralization [3,13], suggesting that other properties of the tissues must be altered.

OPN has also been described as a structural element of bone matrix. By acting as a “glue” at the mineral-collagen interface, OPN forms sacrificial bonds that can resist the separation of mineralized collagen fibrils and enhance bone's toughness [4,[14], [15], [16]]. Therefore, the removal of the protein could facilitate crack propagation and impair bone's toughness.

The toughness of bone is significantly reduced in osteopontin deficient (*Opn*^−/−^) mouse bone, compared to healthy bone, which suggests that OPN plays an important role in preventing crack propagation [3,4]. Using quantitative analysis of 2D small angle x-ray scattering (SAXS) spectra, Poundarik et al. revealed that an absence of OPN increases crystal mis-orientation [17,18]. OPN deficient mice also presented an increase in trace elements typically found in mineral substitutions [18]. Taken together, these studies suggest that OPN critically influences the quality of bone – defined as the nanoscale chemistry, structure and organization of the bone matrix – however there is little information available about the impact of OPN on bone tissue at this length scale.

In this study, we use high resolution transmission electron microscopy (TEM) to investigate the influence of OPN deficiency on the structure of the individual collagen fibrils and the organization of both the collagen matrix and mineral phase in bone. To achieve this correlation, we collected serial thin sections of bone tissue from wild type (WT) and *Opn*^−/−^ bone using an ultramicrotome. For both WT and *Opn*^−/−^ bone, one section was subsequently demineralized in ethylenediaminetetradacetic acid (EDTA) to expose the collagen fibrils. This procedure allowed us to image the same region of both mineralized and demineralized tissue in healthy (WT) and *Opn*^−/−^ bone. We first acquired TEM images showing the organization of collagen matrix at the fibrillar level, after the tissue had been demineralized. The mineral crystal orientation from a serial, mineralized section was then assessed by scanning transmission electron nano-diffraction. This technique provided spatially resolved, quantitative, orientation maps of the mineral phase and its orientation in relation to the underlying collagen fibrils and therefore can be used to quantify difference in collagen organization between WT and *Opn^−/−^* bone. We also compare the mineral density, crystal size, crystal orientation and mineral:matrix ratio in healthy and *Opn*^−/−^ bone.

## 2 Materials and methods

All bone samples were taken from female mice from C57BL/6 background. OPN knock-out mice were 8 weeks old (B6.129S6(Cg)-Spp1tm1Blh/J, The Jackson Laboratory) and control Wild Type were between 8 and 10 weeks of age. The bones for all the experiments were taken from the same mice.

### 2.1 Bulk measurements

#### 2.1.1 Quantitative backscattered scanning electron microscopy (qbSEM)

Right tibia from *Opn*^−/−^ and C57BL/6 (WT) mice (*n* = 5/group) were fixed, dehydrated in ethanol solution with increasing concentration (80% 90%, 100% x2, 24 h per concentration) and embedded in polymethyl methacrylate (PMMA; Sigma-Aldrich, USA) [19]. Samples were cut along the cross-section of the diaphysis and polished with an alumina suspension (1 μm, 0.3 μm and 0.05 μm) and ultrasonicated. Each specimen was carbon coated and imaged by SEM using a four quadrant backscatter detector. Imaging was performed with 20 kV accelerating voltage, saturated filament current, 1.5nA probe current measured with a Faraday cup and at a working distance of 12 mm (Evo, Zeiss, Germany). Pure carbon and aluminum standards (Micro-Analysis Consultants, UK) were imaged before and after each sample and with the same imaging conditions and averaged to account for signal variation. The standards were used to calibrate the SEM signal to calcium content [20]. The whole transverse cross-section was used to assess the degree of mineralization reported as mean ± std for each group.

#### 2.1.2 X-Ray diffraction (XRD)

Right humeri (*n* = 5/group) were scanned by wide-angle x-ray diffraction (Rigaku RAPID II, Rigaku Americas Corp, US) at their mid-diaphysis. Scanning was performed at 40 kV voltage, 30 mA current and 1.2 kW transmission power. The X-ray source was copper with λ = 1.5418 Å. Each sample was tested for 60 min while spinning around its longitudinal axis at 1 deg/sec. The background intensity was subtracted and the diffraction pattern was further analyzed in PDXL 2 (version 2.0.3.0) and 2DP (version 1.0.3.4) software. The full-width at half-maximum (FWHM) for the peak at diffraction angle 2θ = 26° was extracted and the average length corresponding to (002) plane for all the crystals (intra and extra fibrillar) for the scanned area was calculated with Scherrer equation:

$$B=\frac{k\lambda }{L\cos\theta }$$

Where k is the shape factor taken as 0.9, B is the crystal length, λ is the wavelength of X-ray, and L is the FWHM for the peak at diffraction angle 2θ. Results were reported as mean ± std for each group.

#### 2.1.3 Thermogravimetric analysis

The diaphysis of left humeri (*n* = 5/group) were used to measure the weight percentage of mineral to the organic matrix in bone by thermogravimetric analysis (TGA). The samples were heated from 25 °C to 700 °C at a constant rate of 10 °C/min under air flow (Q50, TA Instruments, US). In this analysis loss of moisture, organic content and carbonated mineral was considered to occur up to 200 °C, between 200–600 °C and above 600 °C, respectively. To eliminate the effect of prior water content in the bone, the mineral to matrix ratio was computed by normalizing the organic and mineral mass to water mass at 200 °C based on the following equation:

$$Mineral\;to\;matrix\;ratio=\frac{\frac{mass_{600^{\circ }C}}{mass_{200^{\circ }C}}}{1-\frac{mass_{600^{\circ }C}}{mass_{200^{\circ }C}}}$$

Results were reported as mean ± std for each group.

#### 2.1.4 Small angle X-Ray diffraction

Small-angle x-ray scattering (SAXS) was used to quantify bulk mineral alignment -which corresponds to the orientation of collagen fibrils- in the bone matrix. SAXS data were acquired using a Bruker Nanostar-U instrument (Bruker, MA, US) operated at 50 KV and 24 mA. The instrument uses spot-focus mode with a beamstop diameter of 4.3 μm. This system was equipped with a Bruker Hi-Star multi-wire proportional counter SAXS detector operating in 1024 × 1024 pixel mode. A piece of femoral mid-shaft from the *Opn^−/−^* and WT group (*n* = 1/group) were used for SAXS data collection. Each bone was cut along the frontal plane through the femur axis, such that only one side of the cortex was exposed to the x-ray and the scattering signal to noise ratio was maximized. Three non-overlapping points were scanned in the mid-diaphyseal cortex for 30 min. In a post-processing analysis, the intensity data for each point was integrated in the range of 2θ=0–2.8° (along the radial direction) and plotted as a function of the azimuthal scattering angle in Datasqueeze 3.0.14 (courtesy of Dr. Paul Heiney, University of Pennsylvania). To find the degree of the alignment, ρ parameter was used, which accounts for the x-ray scattering from the aligned fibrils divided by the total amount of the scattering (aligned and randomly oriented) in each spot [21]. ρ varies in the range of 0–1, with 0 representing a totally random and 1 indicating a perfectly aligned collagen fibril structure.

### 2.2 Nanostructural analysis

#### 2.2.1 Bone sample preparation

The diaphysis of the left tibia of two Osteopontin-deficient mice (*Opn^−/−^*) and two wild type mice (WT) from C57BL/6 background were sectioned using a low-speed diamond saw (Isomet, Buehler, USA) to cylindrical segments of roughly 1 mm in length and cut along their length into six quadrants. The samples were ground on 4000 grit sand paper (MicroCut P4000, Buehler, USA) to a thickness below 300 µm. The samples were then washed in deionized water, high pressure frozen (Leica EMPACT2, Leica Micro systems, Germany), and freeze substituted over a period of 4 days in a solution of 3% glutaraldehyde, 1% Osmium tetroxide and 0.5% uranyl in methanol (EMAFS, Leica Micro systems, Germany). These methods prompt the formation of amorphous ice that is subsequently replaced by organic solvents at low temperatures, ensuring close-to-native preservation of mineral morphology, crystallinity and collagen structure [22,23]. The substituted samples were washed in acetone and embedded in epoxy resin dissolved in acetonitrile (Quetol 651, Agar Scientific, UK) over a period of 7 days. The resin:acetonitrile ratio was gradually increased every 24 hrs. with resin concentration of 25%, 50%, 75% and 100% without adding a cross-linking agent, in order to reduce the resin's viscosity and favor the diffusion of the resin in the dense bone material. The samples were then transferred to 100% resin solution with cross-linking agent, stored under vacuum for 6 h prior the final embedding for 48 h at 60 °C.

### 2.3 Sectioning

The embedded samples were trimmed with a glass knife before sectioning with a diamond knife (Diatome AG, Austria) on an Ultramicrotome (Ultracut Reichert, Austria). For each sample, two 90 nm-thick serial sections were collected on 200 mesh copper grids coated with a thin carbon film and air dried at 37 °C for 1 h. Focused ion beam (FIB) lift-out of ultrathin TEM sections was used to confirm that the disorganized tissue-features seen in the *Opn*^−/−^ tissues were not generated by mechanical damage generated during the ultramicrotomy process (Helios NanoLab 600 DualBeam FIB-SEM, FEI, USA). Critically, this technique is site-specific and the SEM images of the trenches milled for lift-out of TEM lamellae unambiguously show the site at which the tissue was milled from(Figure SI2). The specimen was coated with approximately 10 nm of carbon and a 20 × 4 µm area of interest was protected by depositing a 2 µm thick platinum layer. During ion milling, the FIB-SEM was operated at 30 kV with beam currents ranging from 3 nA for coarse milling down to 30 pA for final polishing and thinning to less than 100 nm to achieve an electron-transparent section. The ion-milled lamellae were subsequently imaged by in a JEOL 2100 Plus TEM operated at 200 kV under bright-field conditions. All TEM sections were taken from the middle of the bone diaphysis. No evidence of remnant of woven bone and calcified cartilage were observed as previously reported in Balb/C mice [24] and rats [25].

### 2.4 Demineralization

**Fig. 1:**
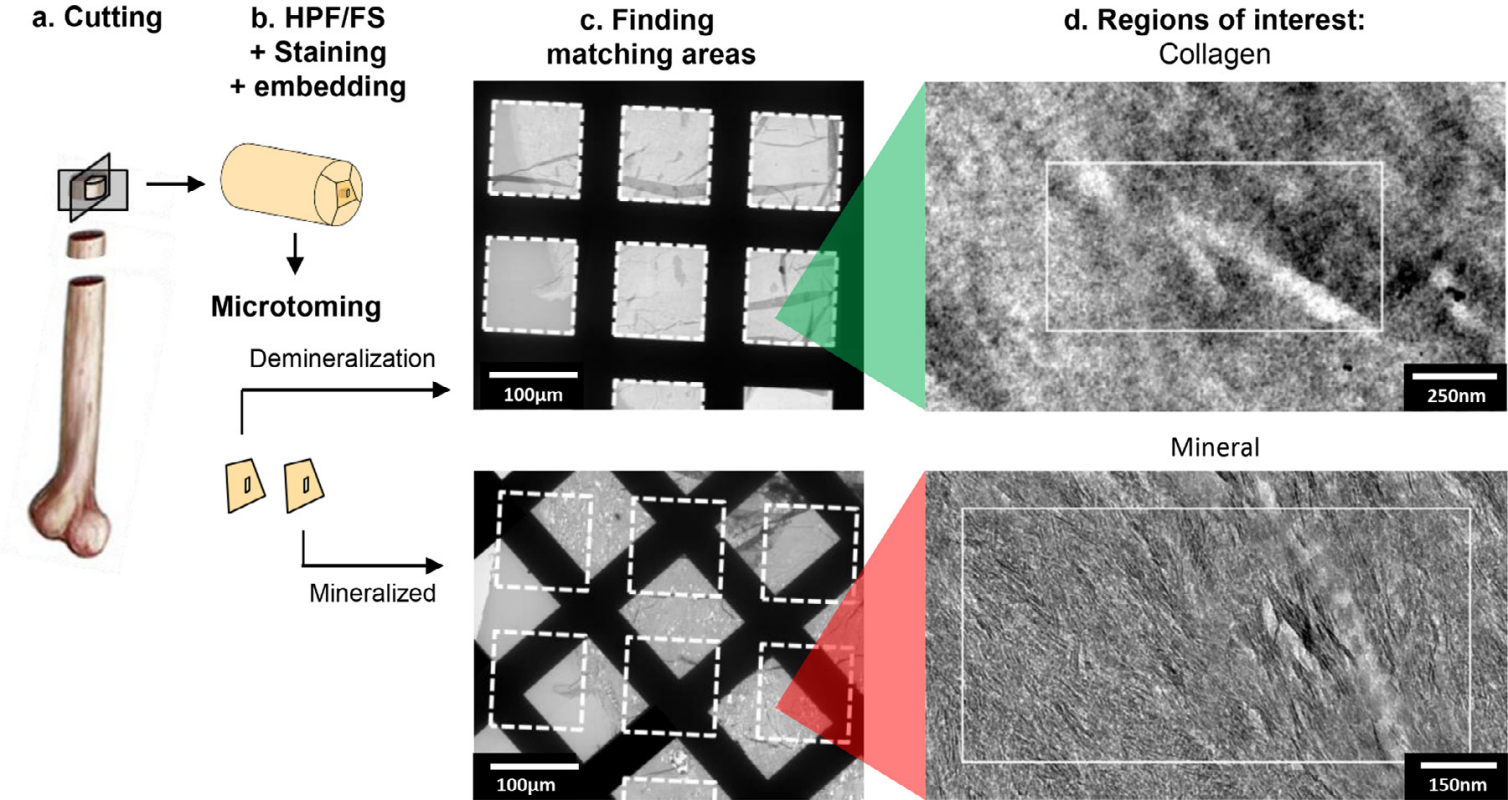
Protocol used to reveal the collagen fibrils and correlate between the mineral and collagen structure/alignment in the bone tissue. Note the image “Finding matching areas” is rotated ~ 45° anticlockwise from row 1 to 2. In order to image both the collagen matrix and mineral phase within the same region of tissue, we studied two serial sections of bone tissue, which were prepared using ultramicrotomy. (a) The diaphysis of the tibia of two Osteopontin-deficient mice (*Opn*^−/−^) and two wild type mice (WT) from C57BL/6 background were sectioned using a low-speed diamond saw into cylindrical segments of roughly 1 mm in length and cut along their length into six quadrants. (b) The bone sections were processed by HPF/FS, bulk stained with a solution of 1% osmium tetroxide and 0.5% uranyl acetate and embedded in an epoxy resin. (c) Two successive ultrathin (70 nm) sections of the bone tissue were cut using an ultramicrotome and collected on two separate TEM grids. One of the ultra-thin sections of the bone tissue was demineralized with EDTA. The area of interest from the demineralized section were manually registered to the mineralized section (dashed squares). (d) Matching areas were identified in high resolution TEM images (white rectangles). All area of interest were taken far from the periosteum and the endosteum of the bone. (For interpretation of the references to colour in this figure legend, the reader is referred to the web version of this article.)

One of the sections of each of the WT and *Opn*^−/−^, (refer to Fig. 1 for method) was demineralized on a TEM grid in a 20 µL drop of 5% ethylenediaminetetraacetic acid (EDTA), 2% paraformaldehyde (PFA) in cacodylate buffer, pH 7 for 5 min. After demineralization, the residual EDTA was washed off the sections three times in deionized water for 5 min and the sections were air-dried at 37 °C for 1 h. Three sections per sample, taken at least 10 µm apart, were used to insure the reproducibility of the observations.

### 2.5 Collagen matrix characterization

In order to visualize how OPN affects the collagen matrix organization and structure at the fibrillar level, and the impact of these on mineral organization, we developed a sample preparation protocol which reveals the collagen fibrils (Fig. 1, SI3). Using this procedure, we were able to unravel the effects of OPN deficiency on collagen fibril structure, organization and mineralization.

### 2.6 Transmission electron microscopy

Transmission electron microscopy (TEM) was performed on an FEI Titan 80–300 (FEI Company, Hillsboro, OR) operated at 300 kV. Regions of interest in the middle of the samples’ diaphysis were mapped at low magnification using bright-field TEM (BF-TEM) prior the nano-diffraction measurements. The nano-diffracted beam was obtained in scanning-TEM (STEM) mode with a convergence angle of 0.23 mrad and a probe size of 6 nm diameter, similar to the thickness of apatite crystals in bone tissue (~5 nm). Such a small probe allows the investigation of single or small bundles of crystals in the tissue and therefore provides orientation information on the sub-10 nm scale. To achieve such a small parallel beam, image resolution had to be sacrificed. However, features of interest were still distinguishable (Fig. SI3a) allowing the images and diffraction data to be aligned with the higher resolution BF-TEM images using a SIFT registration algorithm [26] (Fig. SI3c). Because the data is acquired in STEM mode, the scan coils can be used to raster the beam across the sample collecting a diffraction pattern at each point and build up a map. Diffraction patterns were recorded over a 1000 × 500 nm region of the sample with a step size of 10 nm resulting in 5000 diffraction patterns for each region of interest. For each acquisition, the dose rate was 38.7 e/nm^2^/s for a dwell time of 250 ms, equivalent to 9.7 e/nm^2^, which is lower than previous studies on both collagen [27] and mineral [28].

### 2.7 Image analysis

The diffraction patterns were analyzed to detect the (002) peaks characteristic of the long axis of apatite crystals using an in-house script developed in Matlab (R2016b, The MathWorks Inc., Natick MA, USA). First, the center of the diffraction pattern was detected and used to apply a radial Gaussian blur to reduce the noise in the images. A circular mask was applied to isolate the region containing the (002) peaks and segmented using a luminance threshold of 40%. Within this region, the (002) peaks were defined as the largest connected objects. After ensuring the peaks were symmetrical with respect to the center of the diffraction pattern, the direction of the (002) peaks for each nanobeam electron diffraction pattern and an orientation map was obtained. This map was then overlaid with the bright-field images. The distribution of the mineral orientation was plotted and the when a main orientation peak was present, the full width at half maximum (FWHM) was computed as a measure of the disorganization of the mineral crystals. Finding matching areas between mineralized and demineralized sections was performed manually for each of the measured areas, using the canalicular network as registration markers.

## 3 Results

### 3.1 Bulk structural analysis

**Fig. 2:**
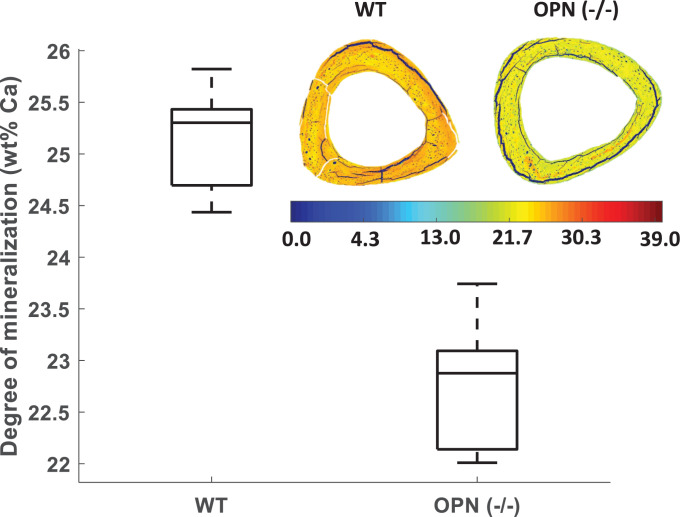
Quantitative backscattered SEM indicates less mineralization in *Opn*^−/−^ bones compared to WT bones at the tissue-level. The box plots represent 25th, median and 75th percentile of the data pooled for all 5 samples. The mineral distribution heterogeneity is not significantly different between the two groups, as illustrated by representative images of the diaphysis cross-sections. (For interpretation of the references to colour in this figure legend, the reader is referred to the web version of this article.)

Bulk analysis of sample from both WT and *Opn^−/−^* groups was performed to assess mineral density, crystal size, crystal orientation and mineral:matrix ratio. Mineral density - measured by quantitative qbSEM - was significantly lower in *Opn*^−/−^ compared to WT tissues (25.23 ± 0.48 vs. 22.74 ± 0.69 wt%Ca, respectively; *p*<0.001, Fig. 2). Analysis of XRD patterns of the bone tissues showed no significant difference between the crystal length (002 axis) in the *Opn*^−/−^ and WT tissues (14.40 ± 0.44 vs. 14.98 ± 1.33 nm, respectively), suggesting no alteration of mineral crystal length in the *Opn^−/−^* tissue. The degree of alignment from SAXS measurements was lower in the *Opn^−/−^* samples when compared to WT samples (ρ_OPN_ = 0.34 ± 0.04 vs. ρ_WT_ = 0.50 ± 0.07, *p*<0.05) showing that collagen fibrils are on average less well aligned with the long axis of the bones. Finally, TGA of the whole bones showed no significant differences in the mineral:matrix ratio between *Opn*^−/−^ and WT tissues (2.99 ± 0.63 vs. 2.99 ± 0.07, respectively). Consequently, a lower mineral density and conserved mineral:matrix ratio suggest that the *collagen content is lower in Opn^−/−^ bones.*

### 3.2 Collagen matrix organization

**Fig. 3:**
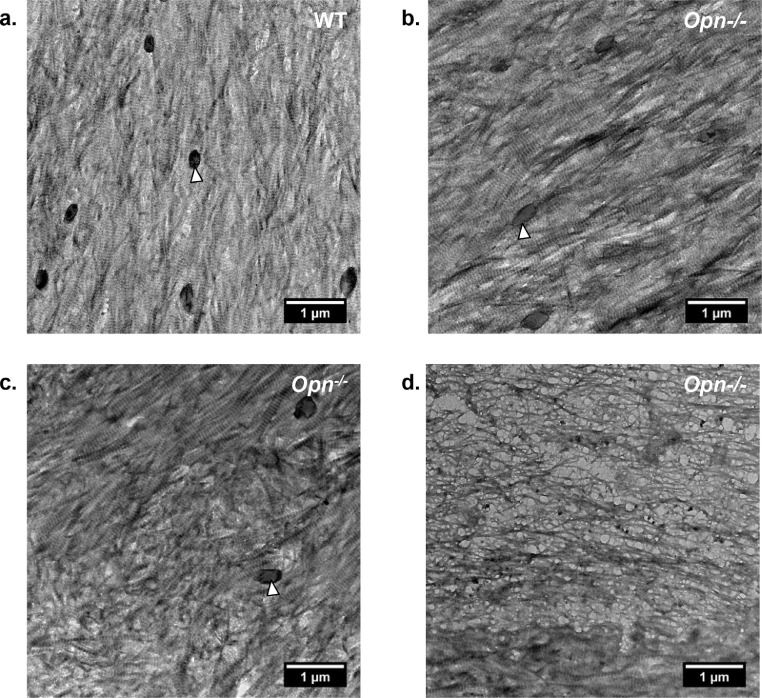
Representative TEM images of demineralized sections of bone tissue of: (a) Wild type and (b-d) *Opn*^−/−^ bone tissue. Most of the ultrastructure of the organic matrix in the *Opn*^−/−^ bone appears minimally disturbed compared to WT tissue (b). *Opn^−/−^* tissue shows pockets of disorganized fibrillar matrix (c) and disorganized non-fibrillar matrix (d). The non-fibrillar matrix in (d) was observed in all Opn^−/−^ specimens, but only seldomly. The black circular features (white arrow heads) are cross-sections of the canalicular network in the bone tissue. (For interpretation of the references to colour in this figure legend, the reader is referred to the web version of this article.)

Fig. 3 and S2 shows the ultrastructure of representative regions of the collagen matrix in WT and *Opn*^−/−^ mouse bone observed along the long axis of the bone. Much of the ultrastructure of collagen matrix was not significantly different in WT (Fig. 3a) and *Opn*^−/−^ tissue (Fig. 3b and S2b) with the orientation of the collagen fibrils aligned principally with the long axis of the tibia. However, in regions of *Opn^−/^* tissue, the collagen matrix was highly disorganized (Fig. 3c and d). Analysis of a serial thin section confirmed that there were no osteocyte lacunae or other major defects near the disorganized tissue. The disorganized collagen matrix could be grouped into two different categories: 1) Fibrils with no clear interfibrillar alignment but showing the characteristic periodic banding pattern (Fig. 3c), and 2) Fibrils with a fibrous mesh-like texture and no characteristic banding pattern and with a lower packing density (Fig. 3d). Both features were observed next to regions of banded collagen (such as observed at the bottom of Fig. 3d). The fibrils’ d-period was not significantly different between WT (*d* = 59.7 ± 1.7 nm) and *Opn*^−/−^ (*d* = 59.5 ± 2.8 nm) tissues, suggesting that OPN deficiency does not significantly alter the structure of assembled collagen fibrils. The collagen exhibited a smaller d-period, possibly because the fibrils in the TEM sections underwent shrinkage as they were dehydrated after demineralization in EDTA. Disorganized areas were not detected in WT bone analyzed from large areas of tissue in multiple samples.

### 3.3 Mineral organization

**Fig. 4:**
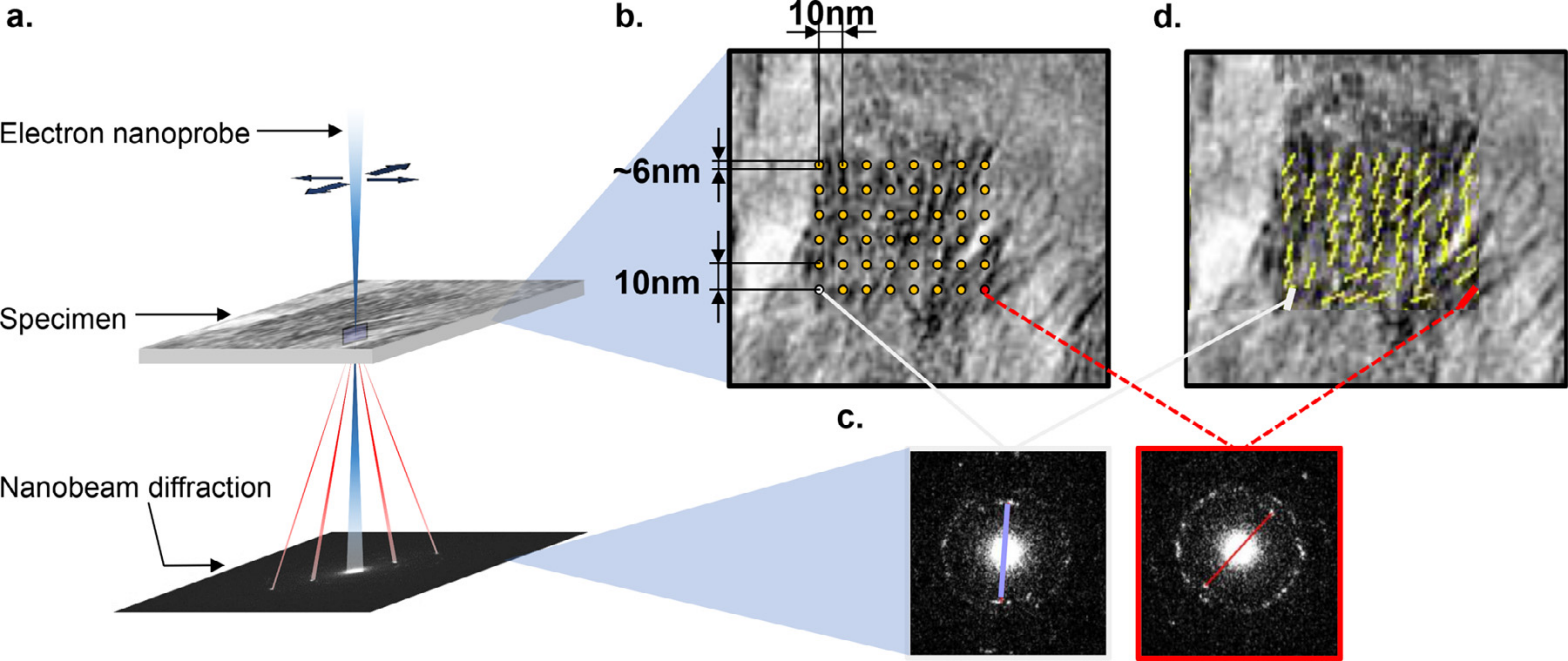
The scanning transmission nanobeam electron diffraction method used to map the mineral orientation within the tissues. In this technique the nanobeam diffraction (NBD) is controlled *via* the STEM imaging mode, which rasters the beam across the sample. Electron diffraction patterns were collected at each point. The smallest dimension of the plate-like crystals was along their thickness that measured ~ 5 nm on average; therefore by using a nano-diffraction beam of ~ 6 nm, we could assess the principle orientation of the individual crystallites (or clusters) by mapping the orientation of the (002) peak of each diffraction pattern. The convergent beam was rastered across the sample within the region of interest (a), and corresponding bright field TEM images (b) and nanobeam diffraction patterns were mapped (c). By placing a mask over the (002) peak in the nanoelectron diffraction pattern, the principle orientation of the mineral crystals was mapped (d). (For interpretation of the references to colour in this figure legend, the reader is referred to the web version of this article.)

To elucidate a role of OPN in mineral alignment, we used scanning transmission nanobeam electron diffraction to measure and map the orientation of the mineral crystals in bone tissue and in relation to the collagen fibril template, using the method described in Fig. 4. This technique maps electron diffraction patterns across the sample. The small electron beam size probes circular areas with a diameter of 6 nm. The mineral platelets in bone crystalize in a hexagonal lattice in an apatitic structure, with the known hexagonal crystal form of HA (*a* = 9.37 Å and *c* = 6.88 Å) [29], and display the characteristic electron diffraction pattern shown in Figure S1. We acquired nanobeam electron diffraction patterns from the mineral crystals and confirmed that the (002) direction of the crystal lattice was aligned with the long axis of the mineral crystals. Therefore, by mapping the orientation of the (002) spot in the nanobeam diffraction pattern, we were able to map the orientation of the long axis of the mineral crystals.

**Fig. 5:**
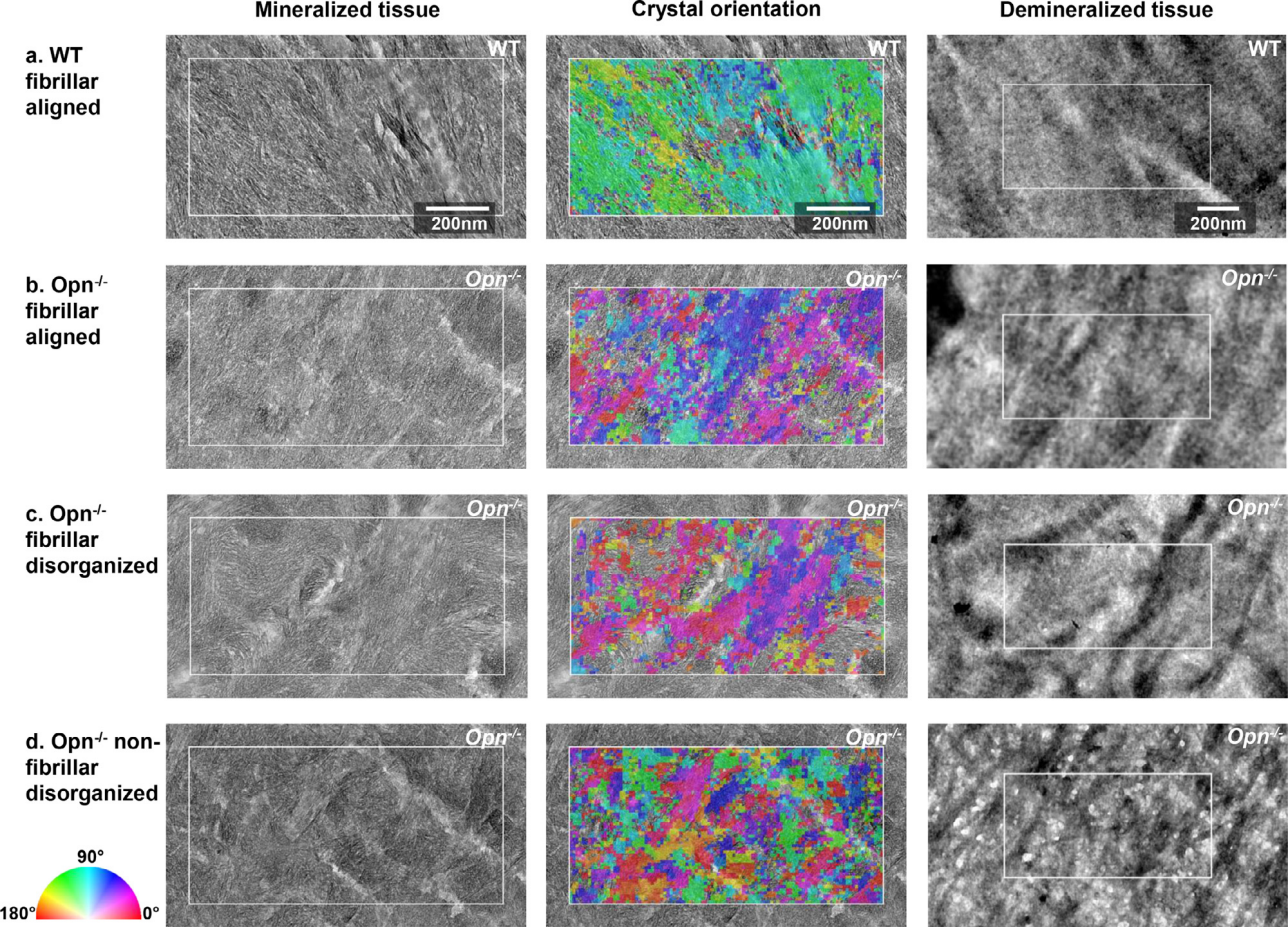
Adjacent sections of mineralized and demineralized bone, showing the crystal orientation (mineralized) and the underlying collagen template (demineralized). The color indicating crystal orientation indicates the orientation of the c-axis (002) plane of the crystals from red = horizontal (0°) to blue = vertical (90°) which have been mapped by NBD. Three characteristic matrix organizations are presented: fibrillar aligned for WT (a) and *Opn*^−/-,^ (b) fibrillar and disorganized (c), and non-fibrillar and disorganized (d). The white rectangle in each image represents the same region of the mineralized and demineralized sections and measure 0.5 × 1 µm^2^. (For interpretation of the references to colour in this figure legend, the reader is referred to the web version of this article.)

**Fig. 6:**
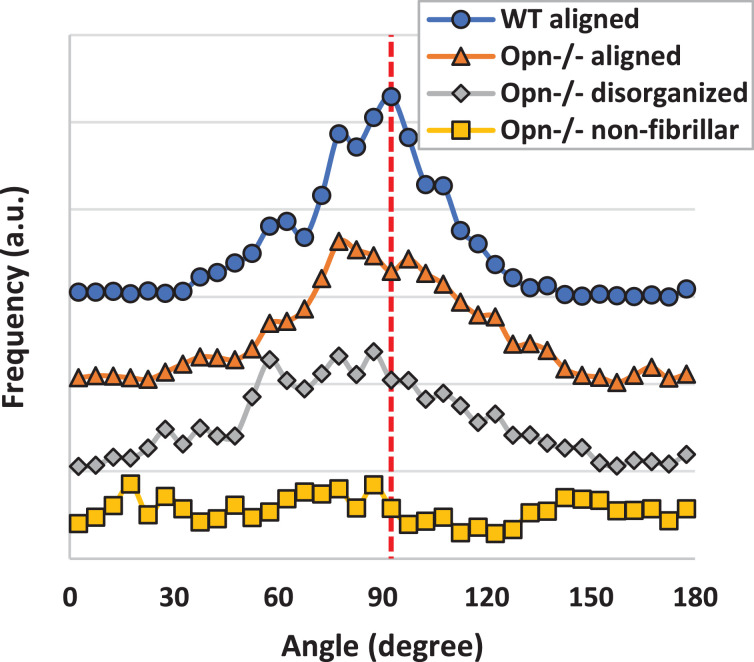
Distribution of the mineral orientation computed form the nano-diffraction measurements. WT tissue exhibits a relatively narrow distribution (FWHM = 36.4°), In areas of Opn^−/−^ tissue where the fibrils are aligned, the orientation of mineral crystal deviate slightly more than in WT (FWHM = 50.7°) bone tissue, while in the disorganized areas, the wide peak indicates a high level of disorganization (FWHM = 62.6°). The data have been centered around 90° for clarity. (For interpretation of the references to colour in this figure legend, the reader is referred to the web version of this article.)

Serial sections of the mineralized and demineralized WT and *Opn*^−/−^ bone tissues are shown in Fig. 5 and the corresponding mineral orientation distribution is shown in Fig. 6. The FWHM of mineral orientation distribution ranged from 36.4° for WT, 50.7° for OPN with aligned fibrils, and 62.6° for disorganized fibrils, indicating that the organization of the mineral was lower in *Opn*^−/−^ than the WT bone tissue. In the WT bone, the mineral crystals exhibited a similar orientation to the underlying collagen fibrils (Fig. 5a), supporting the general consensus that collagen fibrils act as a template for growth of the mineral crystals [30]. Similar observations were made for the *Opn*^−/−^ tissue in regions in which the collagen molecules had assembled into organized fibrils (Fig. 5b). The distribution of mineral orientation exhibited a slightly broader peak when compared to WT tissue (larger FWHM), suggesting that the collagen matrix might be slightly altered. When the fibrils were disorganized, the mineral orientation distribution was clearly affected, as evidenced by the wide peak and large FWHM (Fig. 6). However, the mineral platelets were still aligned along the fibril's long axis (Fig. 5c), which implies that the collagen matrix is altered. When the fibrillar template was absent (Fig. 5d), the orientation of the mineral crystals was disorganized and no peak could be detected in the mineral orientation distribution (Fig. 6). Characteristic regions with poor mineral organization were also observed in *Opn*^−/-^ tissue that had been FIB milled out from the center of the cortex of the diaphysis (Figure SI2).

## 4 Discussion

Even though OPN has been known as a key player in the mineralization of bone tissue, the results of this study shed new light on the function of OPN in formation of the mineralized bone matrix. The findings show that the protein plays a crucial role in the organic matrix formation and structural integrity. Indeed, the lack of OPN in bone clearly affects the collagen matrix, evidenced by poorer tissue organization and the presence of patches of non-fibrillar collagen in the *Opn^−/−^* bone, although the mineral size was conserved. Our results also suggest that the overall collagen content is reduced in bone lacking OPN.

There are no previous reports showing the role of OPN deficiency on collagen matrix structure and organization at the fibrillar level in bone tissue, though alterations to collagen have been reported on in skin, where collagen fibril formation in the deeper dermal layers of a wound-site in skin is affected [31]. In this work [31], the *Opn*^−/−^ mice displayed greater matrix disorganization and altered collagen fibrillogenesis at the wound site, leading to the formation of collagen fibrils with smaller diameter compared to the WT controls. Such alteration occurs only at wound sites, supporting the data shown here that OPN affects fibrillogenesis. Furthermore, *Opn^−/−^* mice do not lose bone after mechanical unloading [32] or hormonal unbalances [33] as in WT mice, which also support the importance of OPN in remodeling. OPN is thought to promote or regulate collagen fibrillogenesis by controlling the adhesion of osteoclasts and osteoblasts to the bone surface during remodeling and to play an important role in both resorption and formation of the collagen matrix [34,35]. The lower mineral density reported here and lower crystal thickness in *Opn^−/−^* tissue [18] could also indicate a lower secretion of collagen and a higher turnover in *Opn^−/−^*. Indeed, OPN has been linked to remodeling activity in bone and tendon [36]. OPN is also heavily recruited during wound healing and remodeling in bone where it binds to the old bone margin to contribute to the cement line formation [37,38]. As anticipated by McKee and Nancy [38], our results suggest that OPN provides, directly or indirectly, a matrix-matrix adhesion mechanism for maintaining the biomechanical integrity of bone tissue. In the absence of OPN, the integrity of the organic matrix has partly deteriorated, resulting in local patches of fibrillar disorganization. Due to its affinity with remodeling cells, OPN might retain these cells in the matrix production, ensuring a proper assembly and alignment of the collagen matrix. Indeed, the presence of OPN favors osteoclast adhesion to mineralized bone *in vitro*, potentially due to the interaction of OPN arginine–glycine–aspartate acid (RGD) ligand to the aνβ3-integrin receptor of osteoclasts [12,39]. A similar mechanism could be anticipated for osteoblast adhesion to mineral crystallites, as integrin signaling is vital to osteoblast function [12].

As shown previously [18], we confirmed, using SAXS, that the global organization of the collagen at the tissue level is poor in the *Opn^−/−^* tissue (*n* = 1 was used here for SAXS analysis, nevertheless our SAXS measurements corroborate these previous SAXS measurements, suggesting that our data are valid). However, our results show that the bulk disorganization measured in *Opn^−/−^* tissue is due to a largely heterogeneous matrix. Indeed, the ultrastructure of OPN deficient tissue taken from the core of the diaphysis of long bones contain regions with both organized and significantly disorganized collagen matrix (quantified in Fig. 6). When fibrillar collagen is formed, mineral formation is not significantly altered in *Opn^−/−^* mice. The size of the mineral crystals and mineral:matrix ratio was conserved between WT and *Opn^−/−^* bones at the tissue level and alignment between mineral and collagen was not significantly altered by the lack of OPN at the fibrillar level. Mineral crystals follow the collagen template organization, with the crystals’ long axis aligned along the long-axis of collagen fibrils. Therefore, the lower degree of alignment measured in bulk samples using SAXS could confirm the heterogeneity of the samples, reflecting the disorganized areas observed at the fibrillar level. This study focuses on the alteration of collagen matrix and its impact on mineral organization. The use high-resolution TEM techniques naturally limits our ability to quantify the extent of the disorganization at the tissue level, however the application of complementary bulk characterization techniques with TEM imaging and quantitative nanobeam electron diffraction mapping has allowed us to shed fresh insight into how *Opn^−/-^* might alter bone's mechanical integrity. Further studies will be necessary to assess the impact of collagen matrix disorganization highlighted here on the micro- and macro-mechanics of bone.

Here we showed, using TEM, that only patches of bone tissue were affected by the lack of OPN. However, OPN is a protein form the SIBLING (small integrin-binding, N-linked glycoprotein) family [40]. These proteins, have structural similarities, including cell attachment/signaling motif, an acidic serine aspartate-rich MEPE-associated motif (ASARM) and multiple post-translational modifications. It is likely that proteins controlling mineralization are redundant [41] and other proteins from the SIBLING family could compensate for the lack of OPN, explaining why the structural integrity of the tissue is generally preserved. It is unlikely that the disorganized areas of the collagen matrix are remnants of unremodeled endochondral bones, as reported for mice femur [24]. Here, multiple observations of large areas over multiple samples were made, and no disorganized areas were detected in WT tibia samples. Such remnants do not seem to be present in the tibia, possibly due to a difference in formation between tibia and femur.

Taken together, our results suggest that OPN plays a significant role in collagen fibrillogenesis, in the organic matrix interfibrillar assembly or in the mineralized fibrils’ cohesion. In *Opn*^−/−^ tissues, the fibrils can still form following the same self-assembly process as in WT bone, as most of the fibrils exhibited the characteristic banding pattern observed in collagen fibrils. However, the fibrillar structure was heterogeneous with several defects. When the fibrillar structure of the collagen was conserved, the mineralization of the tissue was not significantly affected by the lack of OPN. Measurements of mineral orientation revealed that the mineral still grows along the long axis of the fibrils, as long as the fibrillar structure of the collagen template is present, in a similar fashion to that found in WT bone. The disorientation of the mineral crystallites is therefore predominantly due to the misalignment of the collagen matrix. Although the mineral:matrix ratio did not change in *Opn*^−/−^ mice, an overall lower collagen content could be at the origin of a reduced mineralization at the tissue level. A decrease in collagen and mineral content at the tissue level and greater disorganization of the mineralized collagen fibrils could be the cause of the decrease in mechanical properties reported on for OPN deficient bone in the literature.

## Declaration of Competing Interest

The authors declare no competing financial interest.

## Appendix Supplementary materials

Image, application 1
