# Supplementary material for: Osteopontin regulates type I collagen fibril formation in bone tissue
Source: Acta Biomater. 2021 Jan 15;120:194–202. doi: 10.1016/j.actbio.2020.04.040 (PMC7821990; doi:10.1016/j.actbio.2020.04.040)
Supplement: Supplementary file 1 [file mmc1.docx]

**Osteopontin regulates type I collagen fibrils formation in bone tissue**

*Baptiste Depalle1,2*, Catriona M. McGilvery1, Sabah Nobakhti3, Nouf Aldegaither1, Sandra J. Shefelbine3,4, Alexandra E. Porter1*

1 Department of Materials Science and Engineering, Imperial College London, London, UK

2 The Forsyth Institute, Cambridge, MA USA

3 Department of Mechanical and Industrial Engineering, Northeastern University, Boston, MA USA

4 Department of Bioengineering, Northeastern University, Boston, MA USA

Figure SI1: (a) Bright field TEM image of *Opn-/-* tissue and (b) the corresponding selected area electron diffraction (SAED) pattern and (c) a nanobeam electron diffraction patterns taken from the tissue in (a) showing the characteristic diffraction patterns for bone apatite with the known hexagonal crystal form of HA (a = 9.37 Å and c = 6.88 Å). (c) Nano-diffraction pattern for the WT bone tissue.

| a. | b. | |
| --- | --- | --- |
| 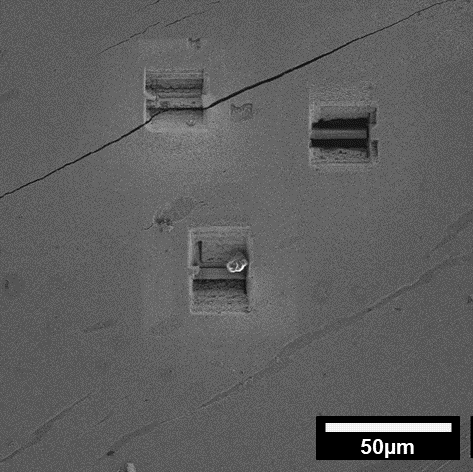 | 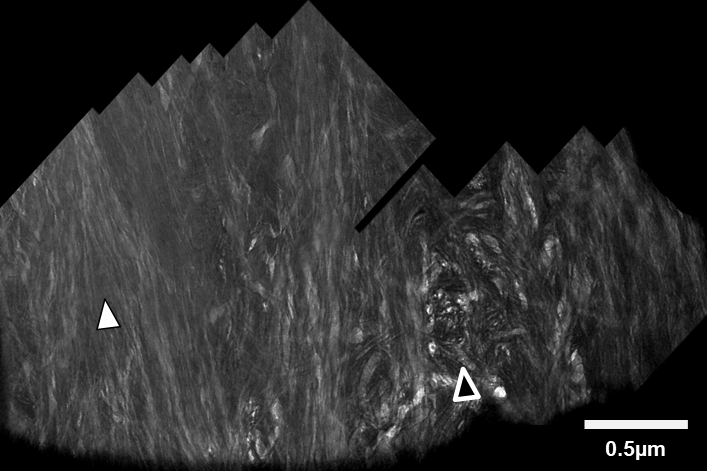 | |
| c. | | d. |
|  | |  |

Figure SI2: (a) Ion-milled sections were extracted from the middle of the diaphysis of a tibia of *Opn^-/-^* mice, far from both the endosteum and periosteum. (b-d) Representative bright field TEM micrographs of ion-milled sections of *Opn^-/-^* bone tissue showing that the disorganization (b and c) and regions of lower packing density (d) within the tissue are inherent characteristics of *Opn^-/-^* and were not produced by the ultramicrotomy process. The large field of view in (b) shows that heavily disorganized patches of tissue (black arrow) are present next to areas of properly formed tissue. Streaks running vertically down the images are curtaining artefacts resulting from the FIB milling process.

| a. | b. |
| --- | --- |
| 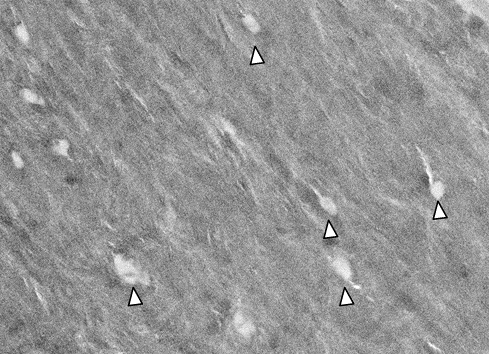 | 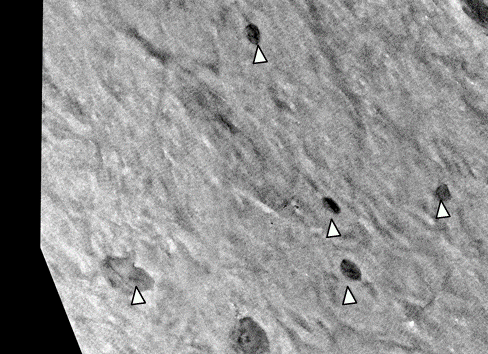 |
| c. | |
|  | |

Figure SI3: Example of TEM images showing the process used to find matching areas. (a) In nano-diffraction mode, image resolution was poor but multiple features of bone tissue were discernable, including the canalicular network (white arrows). These feature were used as markers to manually register adjacent demineralized sections (b) where the canalicular network was also clearly detectable. (c) Finally, high resolution TEM images where automatically registered on images obtained in nano-diffraction mode using a SIFT image registration algorithm.
